# NATIVITY STATUS AND SUCCESSFUL AGING IN LATINX OLDER ADULTS: FINDINGS FROM A CROSS-SECTIONAL SURVEY

**DOI:** 10.1093/geroni/igae098.3157

**Published:** 2024-12-31

**Authors:** Ajla Basic, Mushira Khan

**Affiliations:** Mather Institute, Evanston, Illinois, United States; Mather Institute, Evanston, Illinois, United States

## Abstract

Previous research has underscored the dynamic relationship between health and aging, highlighting the significant influence of social, cultural, and environmental factors on aging outcomes in racial and/or ethnic minority older adults. Building on the extant literature, this cross-sectional study explores the relationship between nativity status and successful aging in community-dwelling Latinx adults 50+, examining whether being born in the U.S. or having immigrated (first-generation immigrant) influences the process of aging well. A total of 141 Latinx older adults (mean age = 62.83; 65.9% female) residing in Chicagoland completed paper surveys. Measures included the successful aging scale, short acculturation scale, well-being indicators including affiliation (social connectedness), autonomy (personal control), and achievement (personal growth), and sociodemographic variables. Findings from multiple regressions revealed that nativity status (U.S. born) moderated the relationship between successful aging and age (F(3, 130)=3.56, p < 0.05), gender(F(3, 133)=5.08, p< 0.05), autonomy (F(3, 134)=2.56, p < 0.05), and self-reported health (F(3,136)=8.83, p < 0.05). These findings suggest that experiences of aging in Latinx older adults can differ depending on whether a person was born in the US or immigrated, indicating the importance of considering factors such as cultural attitudes toward aging, migration and settlement processes, and differential access to resources. Furthermore, cultural norms and expectations surrounding gender roles may be experienced differently by U.S. born Latinx older adults and first-generation immigrants, impacting caregiving, health maintenance, and social support networks. Future research should explore in-depth the mechanisms through which nativity status influences perceptions of aging well in Latinx older adults.

